# Supplementary material for: The Systems Biology Research Tool: evolvable open-source software
Source: BMC Syst Biol. 2008 Jun 29;2:55. doi: 10.1186/1752-0509-2-55 (PMC2446383; doi:10.1186/1752-0509-2-55)
Supplement: Additional file 1 — SBRT Archive. An archive of the current version of the Systems Biology Research Tool. [file 1752-0509-2-55-S1.zip › sbrt-1.4.0/doc/developers_guide/api/sbrt/kernel/math/algebra/package-tree.html]

sbrt.kernel.math.algebra Class Hierarchy


|  |  |  |
| --- | --- | --- |
| **Overview** \| **Package** \| Class \| Use \| **Tree** \| **Deprecated** \| **Index** \| **Help** | |  |
| **PREV**   **NEXT** | **FRAMES**    **NO FRAMES**     **All Classes** |  |


---


## Hierarchy For Package sbrt.kernel.math.algebra

**Package Hierarchies:**: All Packages

---

## Class Hierarchy

- java.lang.Object
  - sbrt.kernel.math.algebra.**AbstractMdMatrix**<R,C> (implements sbrt.kernel.math.algebra.DoubleMatrixBuilder<R,C>)
    - sbrt.kernel.math.algebra.**AbstractSmdMatrix**<R,C> (implements sbrt.kernel.math.algebra.SmdMatrix<R,C>)
      - sbrt.kernel.math.algebra.**HashMatrix**<R,C>- sbrt.kernel.math.algebra.**RowOrTreeMatrix**<R,C>- sbrt.kernel.math.algebra.**TreeMatrix**<R,C>
    - sbrt.kernel.math.algebra.**HashLinearSystemSolution**<V> (implements sbrt.kernel.math.algebra.LinearSystemSolution<V>)- sbrt.kernel.math.algebra.**IndexPair**<R,C>- sbrt.kernel.math.algebra.**MatrixElement**<R,C,E>- sbrt.kernel.math.algebra.**MatrixMultiplier**- sbrt.kernel.math.algebra.**MatrixTransposer**- sbrt.kernel.math.algebra.**MatrixWrap**<R,C,E> (implements sbrt.kernel.math.algebra.Matrix<R,C,E>)
                - sbrt.kernel.math.algebra.**DoubleMatrixWrap**<R,C> (implements sbrt.kernel.math.algebra.DoubleMatrix<R,C>)
                  - sbrt.kernel.math.algebra.**SparseDoubleMatrixWrap**<R,C> (implements sbrt.kernel.math.algebra.SparseDoubleMatrix<R,C>)
                - java.lang.Throwable (implements java.io.Serializable)
                  - java.lang.Exception
                    - java.lang.RuntimeException
                      - sbrt.kernel.math.algebra.**IllegalFreeVariableException**

## Interface Hierarchy

- sbrt.kernel.math.algebra.**LinearSystemSolution**<V>- sbrt.kernel.math.algebra.**LinearSystemSolver**<V>- sbrt.kernel.math.algebra.**Matrix**<R,C,E>
      - sbrt.kernel.math.algebra.**DoubleMatrix**<R,C>
        - sbrt.kernel.math.algebra.**DoubleMatrixBuilder**<R,C> (also extends sbrt.kernel.math.algebra.MatrixBuilder<R,C,E>)
          - sbrt.kernel.math.algebra.**SmdMatrix**<R,C> (also extends sbrt.kernel.math.algebra.SparseDoubleMatrix<R,C>)
          - sbrt.kernel.math.algebra.**SparseDoubleMatrix**<R,C>
            - sbrt.kernel.math.algebra.**SmdMatrix**<R,C> (also extends sbrt.kernel.math.algebra.DoubleMatrixBuilder<R,C>)
        - sbrt.kernel.math.algebra.**MatrixBuilder**<R,C,E>
          - sbrt.kernel.math.algebra.**DoubleMatrixBuilder**<R,C> (also extends sbrt.kernel.math.algebra.DoubleMatrix<R,C>)
            - sbrt.kernel.math.algebra.**SmdMatrix**<R,C> (also extends sbrt.kernel.math.algebra.SparseDoubleMatrix<R,C>)

---


|  |  |  |
| --- | --- | --- |
| **Overview** \| **Package** \| Class \| Use \| **Tree** \| **Deprecated** \| **Index** \| **Help** | |  |
| **PREV**   **NEXT** | **FRAMES**    **NO FRAMES**     **All Classes** |  |


---
